# Inunction in Scarlatina

**Published:** 1854-02

**Authors:** 


*Inunction in Scarlatina.* By Dr. Walz.—British and Foreign Medico-Chirurgical Review. Dr. Walz (Schmidt's Jahrbuch, April,) has employed, after the manner of Schneeman, frictions with fat in 74 patients with scarlatina; all were cured. In 69 cases there were no desquamation; in 4 cases there was secondary dropsy, which was easily cured in one case by diaphoretics, in three by sulphur. The same treatment has been employed in measles.—[*Charleston Med. Jour. and Review.*

